# Chronic exercise remodels the lysine acetylome in the mouse hippocampus

**DOI:** 10.3389/fnmol.2022.1023482

**Published:** 2022-10-28

**Authors:** Ping Qian, Feifei Ma, Wanyu Zhang, Dingding Cao, Luya Li, Zhuo Liu, Pei Pei, Ting Zhang, Shan Wang, Jianxin Wu

**Affiliations:** ^1^Beijing Municipal Key Laboratory of Child Development and Nutriomics, Capital Institute of Pediatrics, Beijing, China; ^2^Graduate School of Peking Union Medical College, Beijing, China; ^3^Beijing Tongren Hospital, Capital Medical University, Beijing, China

**Keywords:** exercise, post-translational modification, acetylation, hippocampus, adult neurogenesis

## Abstract

Physical exercise benefits hippocampal function through various molecular mechanisms. Protein acetylation, a conserved and widespread post-translational modification, is involved in the synaptic plasticity and memory. However, whether exercise can change global acetylation and the role of acetylated proteins in the hippocampus have remained largely unknown. Herein, using healthy adult mice running for 6 weeks as exercise model and sedentary mice as control, we analyzed the hippocampal lysine acetylome and proteome by Liquid chromatography-tandem mass spectrometry. As a result, we profiled the lysine acetylation landscape for the hippocampus and identified 3,876 acetyl sites and 1,764 acetylated proteins. A total of 272 acetyl sites on 252 proteins were differentially regulated by chronic exercise, among which 18.58% acetylated proteins were annotated in mitochondria. These proteins were dominantly deacetylated and mainly associated with carbon-related metabolism, the Hippo signaling pathway, ribosomes, and protein processing. Meanwhile, 21 proteins were significantly expressed and enriched in the pathway of complement and coagulation cascades. Our findings provide a new avenue for understanding the molecular mechanisms underlying the benefits of exercise for hippocampal function and can contribute to the promotion of public health.

## Introduction

Physical inactivity is increasing globally and increases the risk of cognitive decline, whereas exercise benefits brain function (Duzel et al., 2016). Exercise not only boosts memory performance of healthy individuals, but is also an effective strategy to delay or prevent the onset of multiple neurodegenerative diseases, such as cerebrovascular diseases and Alzheimer’s disease (Nay et al., 2021). As a striking example, aged adults participating in 1-year endurance exercise exhibited 2% increase in hippocampal volume, which was accompanied by improved memory function (Erickson et al., 2011). Moreover, evidence from rodents indicates that increased gray matter volume induced by exercise tends to occur in the hippocampus (Maass et al., 2015; Duzel et al., 2016), prefrontal (Erickson et al., 2014) and entorhinal cortex (Whiteman et al., 2016), but not the thalamus or caudate nucleus (Erickson et al., 2011). The hippocampus, an essential region involved in learning and memory, is highly plastic to exercise and has, therefore, been extensively studied. Abundant evidence demonstrates that exercise remodels the structure and function of the hippocampus by enhancing neurogenesis, accelerating new neuron maturation and promoting angiogenesis (O’Callaghan et al., 2009; Marlatt et al., 2012; Cooper et al., 2018). However, in-depth knowledge into the molecular mechanisms underlying the protective effects of exercise on hippocampal function remains elusive.

Proteins are precisely controlled by numerous regulatory processes. Reversible post-translational modifications (PTMs) provide an elegant mechanism to dynamically regulate protein function (Walsh et al., 2005; Salomon and Orth, 2013). Lysine residues can covalently react with diverse substrates to produce PTMs, such as methylation, acetylation, ubiquitination, succinylation, and sumoylation (Sabari et al., 2017). Lysine acetylation is a conserved and widespread PTM, whose homeostasis is maintained by well-tuned balance of lysine acetyltransferases (KATs) and lysine deacetylases (KDACs, also known as HDACs). By regulating protein stability, enzyme activity, subcellular localization and protein-protein/DNA interactions, protein acetylation plays essential roles in protein functions and various cellular processes (Narita et al., 2019). Increased histone acetylation, which activates gene transcription, is mostly thought to be associated with the synaptic plasticity and memory. Regular swimming exercise enhanced hippocampal H3K9, H4K5, and H4K12 acetylation levels and ameliorated isoflurane-induced memory impairment in mice (Zhong et al., 2016). Exercise modalities that improved the memory of aged rats modified H3K9 acetylation at the c-Fos promoter (de Meireles et al., 2019). Recently, the role of non-histone acetylation has also been valued. It is reported that reduction of tau acetylation ameliorated tau-induced memory deficits (Min et al., 2015), and mutations preventing calmodulin acetylation impaired hippocampal synaptic plasticity (Zhang et al., 2021). Furthermore, in a mouse model of Alzheimer’s disease, exercise markedly suppressed acetylation levels of 8-oxoguanine DNA glycosylase and MnSOD, thereby increasing mitochondrial DNA repair capacity and improving cognitive function (Bo et al., 2014). However, Koltai et al. (2011) found that 6 weeks of chronic exercise did not alter the total acetylation level in the rat hippocampus. Therefore, whether exercise could induce global acetylation changes and the roles of acetylated proteins in the hippocampus have remained largely unknown.

In this study, using affinity enrichment and Liquid chromatography-tandem mass spectrometry (LC-MS/MS) analysis, we verified that chronic exercise could alter the acetylation landscape in the hippocampus. We then quantitatively analyzed the acetylome to clarify the roles of acetylated proteins as a bridge between exercise and hippocampus-dependent cognitive function. This study not only enriches the multi-omic database (Sanford et al., 2020), but also furthers our understanding of exercise physiology and helps promote public health.

## Materials and methods

### Experimental animals

Male C57BL/6J mice aged 8 weeks (20–22 g) were purchased from Charles River Laboratory. After a week of habituation to the new environment, mice were randomly divided into two groups: the chronic exercise (Ex) and the sedentary control (Con). All mice were fed *ad libitum* in temperature- and humidity-controlled condition (22 ± 2°C) and maintained on a 12/12-h light/dark cycle (light on at 6:00 am). Body weight was measured biweekly. All animal procedures were conducted during rodents’ nocturnal phase (6:00 p.m. to 12:00 p.m.). The experiments were in strict accordance with the institutional ethical guidelines and approved by the Ethics Committee on Animal Care and Use of the Capital Institute of Pediatrics (DWLL2021015).

### Chronic exercise protocol

**FIGURE 1:**
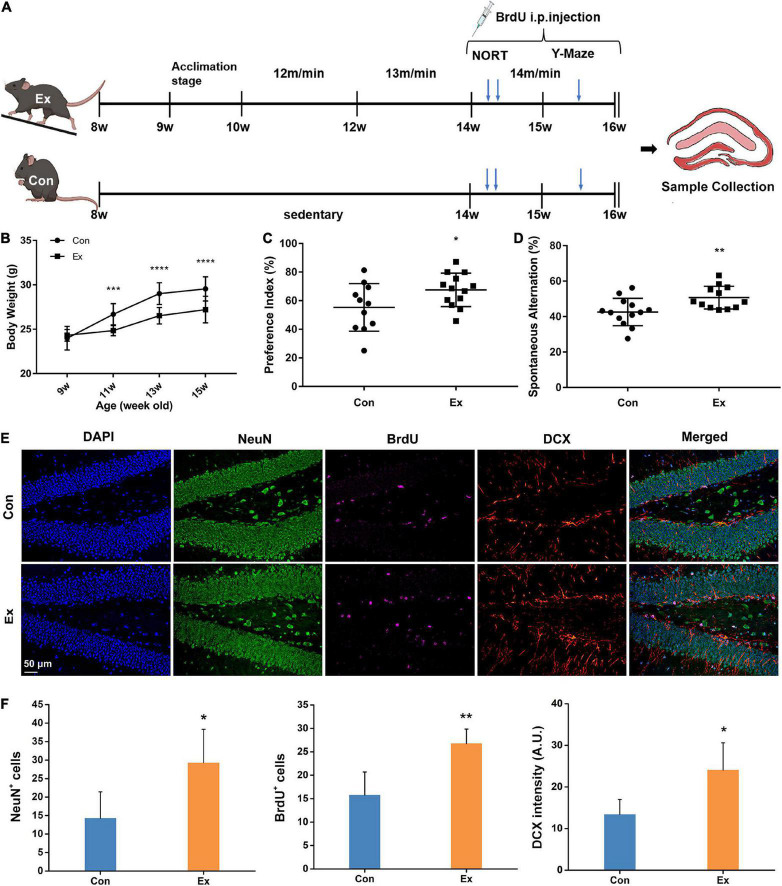
Experimental design and response to chronic exercise. **(A)** Schematic diagram of moderate-intensity chronic exercise model. After habitation to the new environment, mice in Ex group received chronic exercise containing acclimation stage (1 week) and training stage (6 weeks), while the Con mice remained in the same room without access to the treadmill. In a separate cohort, novel object recognition test (NORT) and Y-maze test were performed in the fifth or sixth week of the training stage. BrdU was intraperitoneally injected for last 14 days before scarification. **(B)** Physical response to chronic exercise. Chronic exercise significantly inhibited the growth-related gain of body weight (*n* = 13). ***p < 0.001, ****p < 0.0001 Ex vs. Con at each time point, two-way ANNOVA test. **(C,D)** Cognitive response to chronic exercise assessed by NORT and Y-Maze test (*n* = 11–13). Compared with the control, chronic exercise significantly improved novel object recognition memory (*p* = 0.0458) and spatial working memory in the Ex group (*p* = 0.009). Each dot represents one mouse. **(E)** Representative immunofluorescence images of NeuN, BrdU, and DCX for hippocampal adult neurogenesis. Scale bar = 50 μm. **(F)** Quantitative analysis of NeuN, BrdU, and DCX cells in the hippocampus. Compared with the Con (*n* = 6), Ex mice (*n* = 6) had more NeuN^+^ (*p* = 0.041), BrdU^+^ (*p* = 0.0092), and DCX^+^ cells (*p* = 0.0293) in the dentate gyrus of the hippocampus. For panels **(C,D,F)**, data are presented as mean ± SEM, *p < 0.05, **p < 0.01 Ex vs. Con were calculated by unpaired Student’s *t*-test. Con, control; Ex, exercise; NeuN, neuronal nuclei; BrdU, 5-bromo-2′-deoxyuridine; DCX, doublecortin.

As moderate-intensity aerobic exercise is supposed to benefit the cognitive function both in physical and pathological conditions (Prakash et al., 2015), we adopted an exercise protocol of moderate-intensity (approximately 70% VO_2max_) (Fernando et al., 1993). Animal handling was conducted by the same researcher during every stage: acclimation and training stages. Ex mice ran on a motorized treadmill with 5° incline between 7:00 p.m. and 9:00 p.m. During the acclimation stage, Ex mice adapted to a treadmill for 1 week by training for 10–15 min per day and 5 days/week. Mice started running at 6 m/min and accelerated by 2 m/min every 2 min up to the final speed in each day (Sato et al., 2022; Supplementary Figure 1A). The training stage lasted for 6 weeks, 5 consecutive days per week and 60 min per day. Each training session started with warm-up (speed from 6 m/min increased to the assigned speed as above) followed by running at the assigned speed. The assigned speed was 12 m/min for the first 2 weeks and added 1 m/min biweekly (Figure 1A). Electrical stimuli of 0.6 mA current were set to encourage mice to run at the back of each panel. The running distance for each mouse covered 22 km approximately in the training stage.

The Con mice remained in the same room without access to the treadmills throughout the experiment.

### Behavioral tests

Novel object recognition test (NORT) was used to assess non-spatial memory and performed in the fifth week during the training stage (Figure 1A). This experiment was conducted in a 40 × 40 cm open field, with a video camera above to record movements. On the first day, mice adapted to the open field for 10 min. 24 h later, each mouse was allowed 5 min to explore two identical objects (O1 and O2) that were positioned 10 cm away from the horizontal and vertical walls. 3 h later, O1 was replaced by a new object with different shape and color (N). The arena was cleaned with 45% alcohol after each mouse. The time exploring each object (T) was monitored and the preference index was calculated to quantify novel object recognition memory: [T_N_/(T_N_ + T_O2_)] × 100.

Y-maze test was used to assess short-term spatial memory and performed in the last week (Figure 1A). Mouse was placed in the end of one specific arm and allowed to freely explore the three arms for 8 min. The sequences into each arm were recorded. A spontaneous alternation was recorded only when a mouse consecutively entered all the three arms. Spontaneous alternation percentage was calculated by the formula: [spontaneous alternations/(total number of arm entries – 2)] × 100.

To avoid the potential influence of behavioral tests on hippocampus, the behavioral tests were performed in a separate cohort of Ex and Con mice (*n* = 13), and inactive mice during the tests were excluded.

### BrdU injection

Six mice from each group without undergoing behavioral tests received intraperitoneal injection of 5-bromo-2′-deoxyuridine (BrdU) (50 mg/kg body weight, Sigma-Aldrich, MO, United States) once a day (1 h before running for Ex mice) for last 14 days (Figure 1A).

### Tissue collection

Twenty four hours after last running, mice were euthanized and killed by cervical dislocation. Mice with BrdU injection were perfused transcardially with 4% paraformaldehyde, and the whole brain was isolated and immersed in 4% paraformaldehyde. Other mice were perfused transcardially with 0.9% saline solution. Hippocampal tissue was rapidly dissected out on ice-cooled surface. We also collected bilateral vastus lateralis and epidydimal fats from the mice that underwent behavioral tests, as their relative mass represented physical response to chronic exercise. All collected tissues were flash-frozen in liquid nitrogen and then transferred to −80°C until use.

### Multiplex immunofluorescence staining

A triple immunofluorescence using rabbit antibodies was performed (Toth and Mezey, 2007). Briefly, paraffin-embedded brain tissues from mice with BrdU injection (*n* = 6) were sectioned into 4 μm-thick slices followed by dewaxing and hydrating. The sections were treated with 0.3% hydrogen peroxide solution, underwent microwave treatment and were blocked in 5% BSA. Subsequently, they were incubated with the primary antibody NeuN (1:1000, Cell Signaling Technology, Danvers, MA, United States) at 4°C overnight and the secondary antibody HRP-conjugated anti-rabbit IgG (ZSGB-Bio, Beijing, China) at room temperature for 1 h. After TSA reaction of AlexaFluor 488-Conjugated Streptavidin (1:50, Invitrogen, Waltham, MA, United States), the sections were treated with microwave for 15 min and cooled. Furtherly, primary antibodies DCX (1:800, Cell Signaling Technology, Danvers, MA, United States) and BrdU (1:500, Abcam, United States) were performed successively on the same section, then the corresponding secondary detections were performed with AlexaFluor 594- and 633-Conjugated Streptavidin (1:50, Invitrogen, United States), as described for NeuN. After the last microwave treatment and rinsing, the sections were stained with DAPI. Images were captured. Immunostained-positive BrdU and NeuN cells were visually counted in each section by two researchers blind to the intervention groups. Intensity of DCX was analyzed by ImageJ.

### Protein extraction, trypsin digestion, and acetylated peptide enrichment

Considering the small volume of a mouse’s hippocampus, we mixed bilateral hippocampus from every two mice as one replicate and adopted three replicates in each group. Process of peptide preparation was detailed elsewhere (Li et al., 2020; Liu et al., 2021). For proteome, each sample was grinded with liquid nitrogen and homogenized in four volumes of lysis buffer (8 M urea and 1% Protease Inhibitor Cocktail). Then they were sonicated three times on ice using a high intensity ultrasonic processor (Scientz), followed by centrifugation at 12,000 *g* at 4°C for 10 min. Finally, the supernatant was collected and quantified for the protein concentration with a BCA kit (Beyotime, Shanghai, China). For trypsin digestion, 100 ug protein from each sample was reduced (5 mM dithiothreitol for 30 min at 56°C) and alkylated (11 mM iodoacetamide for 15 min at room temperature in darkness). After that, the resultant samples were fully digested with trypsin for two times (1:50 trypsin-to-protein mass ratio overnight and 1:100 trypsin-to-protein mass ratio for 4 h). The resulting peptides were further desalted and vacuum-dried.

For acetylome, process of protein extraction and trypsin digestion was similar to the above except that, additional inhibitors (3 μM trichostatin and 50 mM nicotinamide) were also added to the lysis buffer and 2.5 mg protein from each sample was used for trypsin digestion. Acetylated peptides enrichment was necessary. Tryptic peptides were dissolved in NETN buffer (100 mM NaCl, 1 mM EDTA, 50 mM Tris–HCl, 0.5% NP-40, pH 8.0) and incubated with anti-acetyllysine antibody-conjugated agarose beads (PTM BIO, China) at 4°C overnight with gentle shaking. Then the beads were washed four times with NETN buffer and twice with ddH_2_O. The bound peptides were eluted three times from the beads with 0.1% trifluoroacetic acid (Sigma, United States). The eluted peptides were then combined, vacuum-dried and desalted for LC-MS/MS Analysis.

### Liquid chromatography-tandem mass spectrometry analysis

Liquid chromatography-tandem mass spectrometry was performed at PTM Biolab (Hangzhou, China). Desalted peptides were dissolved in 0.1% formic acid and loaded onto a reversed-phase analytical column (25-cm length, 100 μm i.d.). Peptides for proteome were separated with a gradient of solvent B (0.1% formic acid in 100% acetonitrile) from 6 to 24% over 70 min, increasing to 35% in 12 min and to 80% in 4 min, and then holding at 80% for the last 4 min. Peptides for acetylome were separated with a gradient of solvent B from 6 to 22% over 42 min, climbing to 30% in 12 min and to 80% in 4 min, then holding at 80% for the last 4 min. The flow rate was 450 nL/min. Then the peptides were subjected to capillary source (CaptiveSpray) followed by the timsTOF Pro (Bruker Daltonics) mass spectrometry. The electrospray voltage was set at 1.60 kV for proteome and 1.80 kV for acetylome. For both omics, MS spectra with m/z range of 100–1,700 were analyzed. The timsTOF Pro was operated in parallel accumulation serial fragmentation mode. Precursors with charge states 0 to 5 were selected for fragmentation, and 10 MS/MS scans/cycle were acquired. The dynamic exclusion was set to 30 s.

The resulting MS/MS data were processed using MaxQuant search engine (v.1.6.15.0). Tandem mass spectra were searched against the Mus_musculus_10090_SP_20220107.fasta (17097 entries) concatenated with reverse decoy database. Trypsin/P was specified as cleavage enzyme, missing cleavages were allowed up to 2 for proteome and 4 for acetylome. The mass tolerance for precursor ions was set as 20 ppm in first search and 5 ppm in main search. Carbamidomethyl on Cys was specified as fixed modification, and acetylation on protein N-terminal and oxidation on Met were specified as variable modifications. The false discovery rate (FDR) for proteins, peptides, and acetyl sites was adjusted to <1%.

### Post-translational modification correlation analysis

Post-translational modification correlations were achieved by comparing our data with shared PTMs in the PLMD database (Xu et al., 2017). Both newly identified acetyl sites and overlaps with other types of PTMs were analyzed.

### Characteristics of acetyl isoforms analysis

We first performed principal components analysis^1^ to characterize the signatures of Ex and Con groups. Using Gaussian mixture model, we tried to categorize all the quantified acetyl sites into three groups based on their response to exercise. As the biochemical preference of some enzyme to given substrates may be partially determined by neighboring conserved sequences around the modified site, we paid attention to the motifs of acetyl sites using iceLogo (Colaert et al., 2009). Secondary structure distribution and surface accessibility were also predicted by NetSurfP v1.0 software.

### Subcellular distribution and protein domain analysis

The subcellular distribution was predicted with Wolfpsort.^2^ Protein domains were analyzed with InterPro database.^3^

### Functional enrichment analysis

Gene Ontology (GO) and Kyoto Encyclopedia of Genes and Genomes (KEGG) database were used for functional enrichment analysis.

### Protein-protein interaction network

Protein-protein interaction (PPI) network was constructed using STRING database (version 11.0). For differentially acetylated proteins (DAPs), all interactions with high confidence (confidence score > 0.7) were selected and visualized in R package “networkD3.” Using the MCODE plug-in, we identified the three most highly interconnected clusters. We also analyzed the interactions between DAPs and NeuN or DCX, the markers of adult neurogenesis.

### Statistical analysis

All graph values were expressed as mean ± SEM and statistical significance was assessed by unpaired Student’s *t*-test or two-way ANOVA analysis. For MS/MS data, log_2_ transformation of the relative quantitative value of proteins or acetyl sites were first applied to make the data closely following a normal distribution. Then unpaired Student’s *t*-test was used to calculate statistical significance of difference between the two groups. Finally, a two-way Fisher’s exact test was used for domain, GO and KEGG enrichment analysis as detailed elsewhere (Zhang et al., 2019). Statistical significance for motif analysis was assessed using binomial test. For secondary structure distribution and surface accessibility, statistical significance was calculated using Wilcoxon rank sum test. *P*-values < 0.05 were considered statistically significant throughout the study.

## Results

### Physical and cognitive response to chronic exercise

To assess the effect of moderate-intensity chronic exercise on physical fitness, we first surveyed growth-related body weight gain. After running for 2 weeks, the body weight of Ex mice was significantly lower than that of the Con mice (Figure 1B). Six weeks of moderate-intensity exercise also significantly raised the vastus lateralis mass (Supplementary Figure 1B, *p* = 0.0004) and suppressed epididymal fat gains (Supplementary Figure 1C, *p* = 0.0282) in Ex group when compared with the control. To determine whether chronic exercise improved hippocampal function, we performed NORT and Y-maze test. Compared with the Con, Ex mice showed preference for the novel object (Figure 1C, *p* = 0.0458), indicating facilitated recognition of a novel object. In the Y-Maze test, spatial working memory was evaluated with spontaneous alternation, and Ex mice outperformed their counterparts (Figure 1D, *p* = 0.009). In addition, exercise significantly increased the numbers of BrdU^+^ (*p* = 0.0092), NeuN^+^ (*p* = 0.041), and DCX^+^ (*p* = 0.0293) neurons in the dentate gyrus, indicating enhanced potential of adult neurogenesis (Figures 1E,F). In summary, these results indicate that 6 weeks of moderate-intensity chronic exercise were beneficial to both physical fitness and cognitive function.

### Identification of acetylation landscape in the mouse hippocampus

**FIGURE 2:**
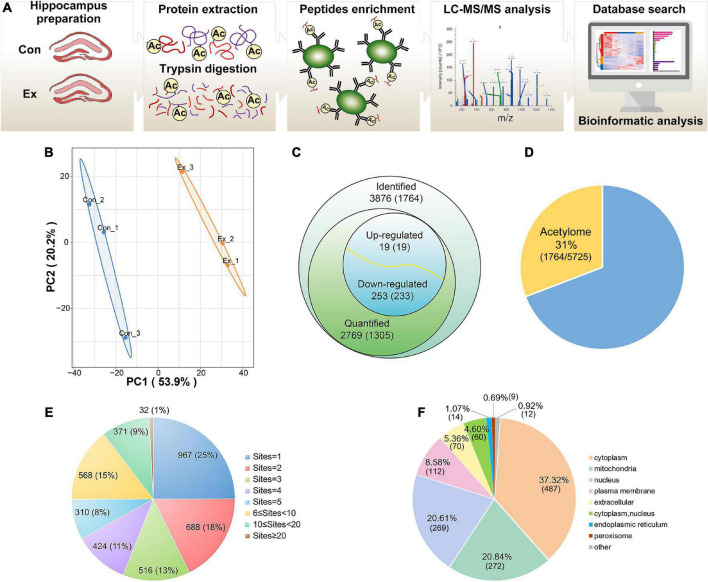
Profiling lysine acetylation proteome in hippocampus. **(A)** Mass spectrometry workflow for identification and quantitation of lysine acetylation in the hippocampus. **(B)** Principal component analysis of acetylome data to distinguish the Ex from the Con. Three biological replicates (bilateral hippocampus from every two mice as one replicate) were performed in each group. **(C)** Global view of acetylated proteins and acetyl sites. Venn diagram showed the number of acetyl sites and the corresponding proteins in brackets. Acetyl sites with a fold change greater than 1.5 or less than 0.667 and with *p*-values < 0.05 were considered as significantly upregulated or downregulated. **(D)** The relative ratio of acetylated proteins identified in the acetylome to proteins identified in the proteome. **(E)** Distribution of the acetyl site occupancy per protein. **(F)** Subcellular distribution predicted for all the quantified acetylated proteins. LC–MS/MS, liquid chromatography-tandem mass spectrometry; PCA, principal component analysis.

To profile the lysine acetylation landscape of the hippocampus in both sedentary and exercise mice, we assessed acetylome using acetyllysine enrichment and LC-MS/MS analysis (Figure 2A). Normalization to total protein was performed to eliminate the effect of protein expression on modification abundance. After evaluation of high-quality MS data (Supplementary Figure 2) and high-degree reproducibility (Figure 2B), we identified 3,876 acetyl sites on 1,764 proteins (Figure 2C and Supplementary Table 1), which constituted 31% of the whole hippocampal proteome (Figure 2D). Up to 75% of the identified proteins harbored more than one acetyl site (Figure 2E). The five proteins with the most acetyl sites were SPTAN1 (32 sites), GOT2 (19 sites), ACO2 (18 sites), MDH2 (17 sites), and CNP (17 sites). In addition, 1,305 proteins with 2,769 acetyl sites were quantified (Figure 2C) and predicted to distribute in virtually every subcellular compartment, with cytoplasm and mitochondria the most dominant localizations (Figure 2F).

### Post-translational modification correlation analysis for acetyl sites

Interactions of PTMs on the same or surrounding lysine sites may affect protein structures and functions, vastly expanding the functional diversity of mammalian proteome (Aebersold et al., 2018). When compared with the PLMD database of mouse lysine acetylation, 716 novel proteins and 2,423 novel acetylation sites were identified in our data (Supplementary Table 2). In addition, some other types of PTMs were found at our identified lysine sites (Supplementary Table 3), such as ubiquitination (962), succinylation (693), and malonylation (540). As acetylation on a specific site influences modifications on its surrounding lysine sites, we also analyzed the known PTMs locating within six residues of our acetylated lysine site (Supplementary Table 4). This PTM correlation analysis mainly indicated acetylation (919), ubiquitination (449), succinylation (399), and malonylation (249). Taking Catalase as an example, the up-to-date data predicted that K449 could be modified by acetylation, ubiquitination, succinylation, malonylation, and glutarylation; the surrounding sites around K480 could be modified by acetylation, succinylation, glutarylation, malonylation, and ubiquitination. These findings have elevated PTMs as a widespread mechanism, among which acetylation and ubiquitination might be the most prevalent. The possibility of crosstalk among distinct types of PTMs on the same or neighboring sites underscores its complexity and physiological relevance in adaption.

### Chronic exercise altered characteristics of acetyl isoforms

**FIGURE 3:**
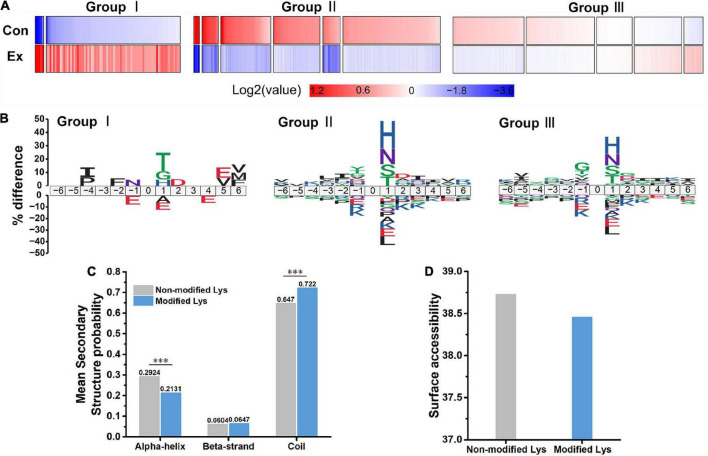
Characteristics of acetyl isoforms in response to chronic exercise. **(A)** Three groups based on the trends of the quantified acetyl sites upon chronic exercise. Group I sites were upregulated by chronic exercise; Group II sites were downregulated in response to exercise; and Group III exhibited unchangeable. **(B)** Predicted amino acid motifs for each group. The motif showed significant (*p* < 0.05, binomial test) amino acid at each site flanking the position 0 lysine. **(C,D)** The conformational tendencies of 272 differentially acetylated sites predicted by protein secondary structures and surface accessibility. Compared with the non-modified lysine residues, the differentially acetylated sites were significantly enriched in coil (*p* = 9.80 × 10^–13^) and depleted in alpha-helical regions (*p* = 1.26 × 10^–11^, Wilcoxon rank sum test), while showed no difference in surface-exposed accessibility (Wilcoxon rank sum test). ^***^p < 0.001.

To explore the overall trends of the quantified acetyl sites upon exercise intervention, 13 clusters generated by a probabilistic clustering algorithm were separated into three groups: Group I sites that were upregulated by chronic exercise; Group II sites that were decreased in response to exercise; and Group III sites that were unchanged (Figure 3A). We performed motif analysis for each group to obtain more insights into their physical properties (Figure 3B) and observed that Group I sites preferred Thr at the +1 position, while both Group II and III sites preferred His and Asn at the +1 position. It seems that acetyl sites in Group I tend to be near acidic amino acids while those in Group II and III tend to be near basic amino acids.

In addition, to uncover the conformational tendencies of differentially acetylated sites (DASs) changed by exercise, we initially defined sites with fold changes (>1.5 or <0.667) and *p* < 0.05 as DASs between Ex and Con groups. As a result, 272 acetyl sites were significantly regulated, including upregulated 19 sites and downregulated 253 sites (Figure 2C and Supplementary Table 5). Compared with the non-modified lysine residues, the DASs were significantly enriched in coil (*p* = 9.8 × 10^–13^) and depleted in alpha-helical regions (*p* = 1.26 × 10^–11^, Figure 3C). In parallel, DASs displayed no difference in surface-exposed accessibility (Figure 3D). These findings suggest that acetyl sites differentially modified by exercise in the hippocampus possibly affect protein function by changing preferences for neighboring amino acids and secondary structure but not surface accessibility.

### Subcellular distribution and protein domain analysis of significantly up and downregulated acetylated proteins

**FIGURE 4:**
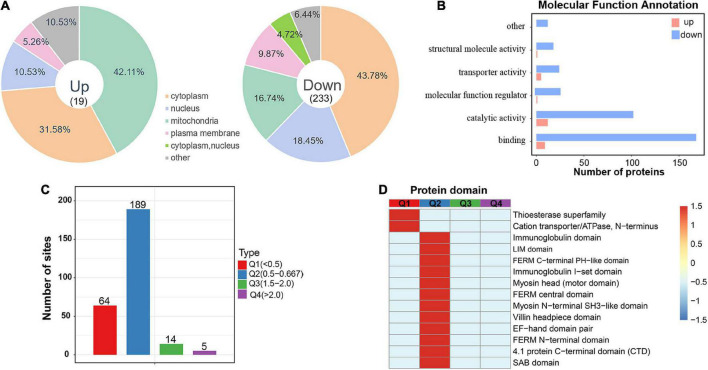
Subcellular location and protein domain analysis of significantly up and downregulated acetylated proteins. **(A)** Subcellular location analysis for significantly up and downregulated acetylated proteins, respectively. **(B)** Molecular function annotation for significantly up and downregulated acetylated proteins. **(C)** Degree of modification of differentially acetylated proteins. **(D)** Protein domain analysis for the Q1–Q4 quantiles in panel **(C)**.

To investigate acetylated proteins in the mouse hippocampus altered by chronic exercise, we found 19 proteins were significantly upregulated and that 233 proteins were significantly downregulated, corresponding to 19 and 253 DASs, respectively (Figure 2C). For the 252 DAPs, their predicted annotation of subcellular distribution (Supplementary Figure 3) was similar to that of the quantified acetylated proteins (Figure 2F). However, there was subtle difference in the subcellular location between up and downregulated DAPs. As illustrated in Figure 4A, upregulated DAPs were mainly annotated in mitochondria, while the downregulated DAPs were most abundant in cytoplasm. Annotation of molecular functions showed that up and downregulated DAPs were significantly enriched in catalytic activity and binding, respectively (Figure 4B). To further clarify DAPs, we divided them into four categories (Q1–Q4) based on the fold-change values (Figure 4C). Based on the domain enrichment analysis, DAPs in Q1 were enriched in thioesterase superfamily members and cation transporter/ATPases, DAPs in Q2 were enriched in other domains, such as immunoglobulin and LIM domains. However, domains were weakly enriched for upregulated acetylated proteins, probably because of limited acetylated proteins in Q3 and Q4 categories (Figure 4D).

### Functional enrichment analysis for differentially acetylated proteins

**FIGURE 5:**
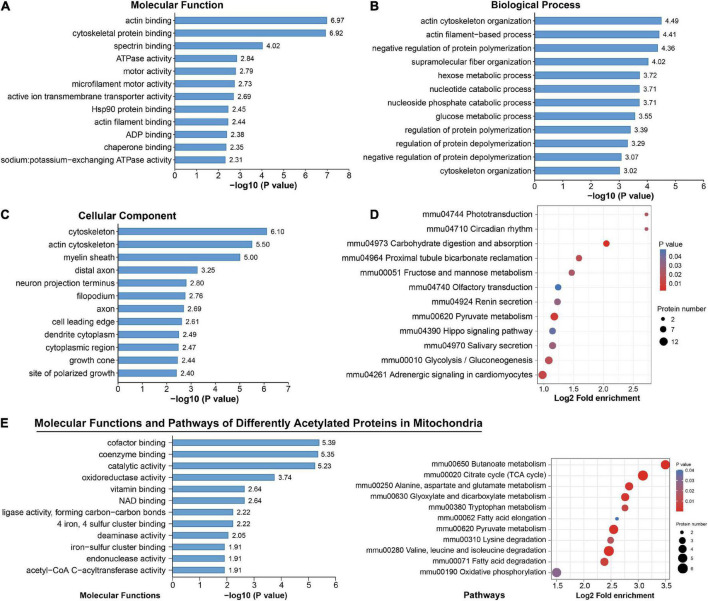
Functional enrichment analysis for differentially acetylated proteins. **(A–D)** Representative Gene Ontology (GO) enrichment analysis (molecular function, cellular component, biological process) **(A–C)** and enrichment analysis of Kyoto Encyclopedia of Genes and Genomes (KEGG) pathways **(D)** of all differentially acetylated proteins. **(E)** Molecular function and KEGG pathways of 47 proteins that were differentially acetylated in mitochondria.

To identify the biological functions of DAPs, we assigned GO annotations (Figures 5A–C). In the cellular component category, acetylated proteins were significantly enriched in cytoskeleton, myelin sheath and axons, which were consistent with their subcellular distribution. Actin cytoskeleton organization, regulation of protein polymerization or depolymerization, and hexose/glucose metabolic process were significantly enriched in the biological process category. According to KEGG pathway analysis, the DAPs were connected to central carbon metabolism, neurodegeneration diseases, synapses, and various signaling pathways (Supplementary Figure 4). Among them, significant alterations clearly highlighted in carbon-related metabolism (carbohydrate digestion and absorption, glycolysis/gluconeogenesis, fructose and mannose metabolism, and pyruvate metabolism) and Hippo signaling pathways (Figure 5D). The DAPs in the pathway of glycolysis/gluconeogenesis included PFK1, ALDOC, TPI, and GAPDH (Supplementary Figure 5A). Those in the Hippo signaling pathway included 14-3-3 gamma and subunits of serine/threonine-protein phosphatase (PPP2R1A, PPP1C, PPP2C) (Supplementary Figure 5B). We noticed that phototransduction, circadian rhythm, olfactory transduction and salivary secretion were also featured by chronic exercise (Figure 5D).

Among these DAPs, there were 18.58% (47) annotated in mitochondria (Supplementary Figure 3), and 39 were downregulated (Figure 3A). These mitochondrial DAPs were predicted mainly binding with cofactor, coenzyme, NAD, iron–sulfur cluster or exerting catalytic activity (Figure 5E). Significant enrichment of major mitochondrial processes included citrate cycle, butanoate metabolism, fatty acid metabolism, amino acid catabolism, and oxidative phosphorylation (Figure 5E). Noteworthily, acetyl-CoA acetyltransferase (ACAT1), which plays a critical role in acetyl-CoA metabolism as well as amino acid catabolism and ketone body synthesis (Haapalainen et al., 2007) was subjected to significantly downregulated acetylation.

Taken together, these results indicate that chronic moderate-intensity exercise may regulate multiple biological functions by modulating relevant protein acetylation levels, possibly mediating the adaption of hippocampus to repeated single exercise challenge and reshaping hippocampal function.

### Analysis of protein-protein interaction networks

**FIGURE 6:**
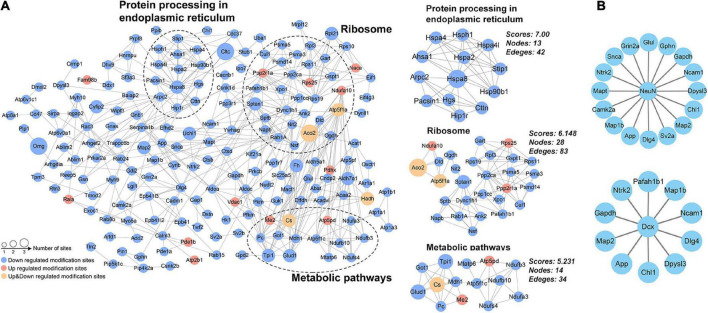
Protein–protein interaction network analysis. **(A)** Network for the differentially acetylated proteins and the three most highly connected subnetworks (metabolic pathways, ribosome, and protein processing in endoplasmic reticulum). **(B)** The differentially acetylated proteins were connected with the markers of adult neurogenesis (NeuN and DCX).

Protein-protein interaction network for DAPs was constructed from 187 proteins with high confidence (Figure 6A). Using the MCODE plug-in, three highly connected clusters were identified: metabolic pathways, ribosomes, and protein processing in endoplasmic reticulum (Figure 6A). The changed acetylation of metabolic and protein processing implied higher demand for energy and translation in Ex mice. Considering adult neurogenesis was positively related to the hippocampal function, we also investigated the relationship between NeuN or DCX and the DAPs. The results showed that several DAPs connected with NeuN or DCX (Figure 6B), suggesting that acetyl modification might play an important role in neurogenesis during exercise.

### Proteomic changes

In addition to serving as a background for quantifying acetylation changes, the proteomic analysis also provided much information concerning exercise physiology. Among 5,725 identified proteins, seven were downregulated and 14 were upregulated using the same standards for DASs. Biological pathway analysis pointed to the only pathway of complement and coagulation cascades. The upregulation of Antithrombin-III and the downregulation of Fibrinogen likely reflected overall inhibition of the coagulation cascade by exercise (Supplementary Table 6). This is in agreement with the plasma proteome of voluntarily running mice (De Miguel et al., 2021). Compared with the acetylome, small changes in the proteome support the hypothesis that acetyl modification of proteins is more sensitive to internal or external stimuli than the protein *per se*, conferring on cells the ability to rapidly react and adapt to environmental demands.

Chronic exercise shifted lysine acetylation toward deacetylation, we therefore analyzed KATs and KDACs in our MS data. Proteomic data covered several KDACs (HDAC 4-6, HDAC 11, SIRT3, SIRT5) and KATs (MORF4L1 and NCOA1), but none was significantly altered. Though SIRT2 had statistical difference, its expression level was equivalent between Ex and Con (Supplementary Table 7).

## Discussion

Physical activity evokes profound response in multiple systems, including improvement in cognitive vitality (Prakash et al., 2015). With comprehensive guidelines on recommended physical activity (Ding et al., 2020) and growing awareness among people of the benefits of physical activity, better understanding of molecular mechanisms underlying physical activity could help improve public health. The Molecular Transducers of Physical Activity Consortium was established to build a molecular map of acute and chronic exercise by means of omics (Sanford et al., 2020). Accordingly, longitudinal multi-omic analyses of human blood components characterized an orchestrated molecular choreography associated with acute exercise (Contrepois et al., 2020). Genome-wide epigenomics revealed DNA hypomethylation-dominant changes in the hippocampus of wild-type mice with chronic exercise (Urdinguio et al., 2021). Lysine acetylation is an evolutionarily conserved PTM and exists across prokaryotes and eukaryotes (Ouidir et al., 2016). Irrespective of its occurrence on histones or non-histones, lysine acetylation is involved in diverse cellular processes relevant to physiology and diseases, such as gene transcription, metabolism, signal transduction, DNA damage repair, and autophagy (Narita et al., 2019). Although several studies have analyzed acetylation changes in the brain associated with exercise, they have mostly focused on histones or individual non-histone proteins (Koltai et al., 2011; Sarga et al., 2013). Here we performed a global acetylome assessment and investigated global acetylation changes in the hippocampus of wild-type mice enduring moderate-intensity chronic exercise. We identified 3,876 acetylated sites on 1,764 proteins, among which 2,423 sites and 716 proteins had not been previously retrieved. Among the quantified 2,769 sites and 1,305 proteins, 272 sites on 252 proteins were differentially regulated by exercise (Figure 2C). These findings indicate that exercise is a major regulator of protein acetylation in hippocampus.

In the present study, a large number of exercise-influenced acetyl sites tended to be near basic amino acids, and likely affected the secondary structure of proteins. Such features of acetyl sites seem common as they have been observed under other conditions, such as calorie restriction (Hebert et al., 2013) and gut microbiota-dysbiosis (Liu et al., 2021). We also noticed that the majority of proteins were multi-acetylated (Figure 2E) and that various other PTMs were located on the same or surrounding lysine sites, including ubiquitination and succinylation (Supplementary Tables 3, 4). Protein ubiquitylation is another major PTM that mediates protein degradation *via* the proteasome complex. A survey of ubiquitylation sites in human cells showed that about one-third of acetyl sites were also subject to ubiquitylation (Wagner et al., 2011). It is easy to imagine that a mixture of PTMs increases chances for interaction. For example, acetylation of K50 in 14-3-3-ε reduced binding to targeted phosphopeptides, while acetylation of K118 plus K123 severely impaired this binding, and triple acetylation of the above sites completely abolished phosphopeptide binding (Choudhary et al., 2009). Additionally, methylation of p53 on K372 promoted the acetylation of K373, resulting in subsequent transcription of p21 and cell cycle arrest (Ivanov et al., 2007). Thus, intricate crosstalk of PTMs can vastly expand the functional diversity of proteins and is worthy of investigation to help understanding hippocampal function.

Acetylated proteins were predicted to distribute across diverse subcellular compartments, not just be confined to the nucleus (Figure 2F and Supplementary Figure 3). This is consistent with previous findings (Choudhary et al., 2009; Sun et al., 2021), indicating their essential roles on non-histone proteins. Focused studies indicate that protein acetylation is a major mechanism in glucose homeostasis (Wang et al., 2010; Yang et al., 2011; Lundby et al., 2012). In a mouse model of gut microbiota dysbiosis-induced depression, the significantly acetylated proteins in hippocampus were primarily related to carbon metabolism (Liu et al., 2021). In human brains, protein acetylation is associated with metabolic pathways, the tricarboxylic acid cycle and neurodegenerative disease (Sun et al., 2021). Similarly, our enrichment analysis found several pathways of carbon-related metabolism (carbohydrate digestion and absorption, glycolysis/gluconeogenesis, fructose and mannose metabolism, and pyruvate metabolism) (Figure 5D). And acetylation of four enzymes responsible for catalysis from fructose-6-phosphate to 1, 3-bisphosphoglycerate in glycolysis (PFK1, ALDOC, TPI, and GAPDH) was downregulated by exercise. Consequently, chronic exercise may influence the hippocampal function by altering the acetylation levels of proteins related to carbon metabolism.

It is common to know that mitochondria are central mediators for energy production and metabolism, and appear to harbor extensive acetylated proteins (Kim et al., 2006; Choudhary et al., 2009). In the current study, 18.58% DAPs were annotated in the mitochondria, functioning in multiple pathways, especially of energy metabolism (Figure 5E; Wang et al., 2010). Intriguingly, most of the mitochondrial DAPs were downregulated. Although some reports indicate that metabolic proteins can be activated upon acetylation (Li et al., 2014), the majority of well-documented cases demonstrate that activity of mitochondrial enzymes is inhibited by acetylation (Lundby et al., 2012; Baeza et al., 2016; Nakayasu et al., 2017). Besides, improper mitochondrial acetylation is detrimental to numerous processes of oxidative and metabolism (Finley et al., 2011; Hirschey et al., 2011). As mitochondria have a central role in aging-related neurodegenerative diseases (Lin and Beal, 2006), whereas exercise is known as a powerful intervention against those partially by regulation mitochondrial dynamic equilibrium (Ringholm et al., 2022), it’s tempting to speculate that exercise-induced deacetylation favors regulation of mitochondrial metabolic pathways toward more efficient fuel utilization, providing evidence for the benefits of exercise on the brain.

Besides metabolic pathways, DAPs were also significantly enriched in the Hippo signaling pathway (Figure 5D) and related with markers of adult neurogenesis (Figure 6B). The Hippo pathway functions in regulating cell growth and proliferation and controls organ size (Fu et al., 2017). Recently, it has been shown to function in neuroinflammation, neuronal cell differentiation, and neuronal death (Cheng et al., 2020). Moreover, we detected acetylated 14-3-3 gamma in this pathway. By regulating their targets, 14-3-3 proteins play a role in neurogenesis, neuronal migration and differentiation, synaptogenesis and dopamine synthesis (Antunes et al., 2022). The biological relevance of acetylation has been investigated in some 14-3-3 isoforms (Choudhary et al., 2009; Aghazadeh et al., 2014; Frontini-Lopez et al., 2021); however, we identified different acetylated site on 14-3-3 gamma. Therefore, the function of acetylated 14-3-3 gamma in Hippo pathway as well as other DAPs with hippocampal neurogenesis during exercise remains to be elucidated.

We noticed that DAPs were also significantly enriched in phototransduction, circadian rhythm, olfactory transduction and salivary secretion (Figure 5D). Alterations in circadian rhythm-related DAPs were unlikely attributed to the artificial disturbance, because we tried our best to control the common housing environment, light time, the fixed time of training and the sampling time. It is reported that physical exercise regulated the autonomic nervous system differentially (Kjaer et al., 1987), reversed age-related vulnerability to retinal damage (Chrysostomou et al., 2016). improved recovery of olfactory function (Wang et al., 2022) and stimulated salivary secretion (Sant’Anna et al., 2019). Hence a whole-body response to exercise possibly occurred, and investigations into the sophisticated and elegant crosstalk between tissues during exercise are of interest for research.

Contrary to the acetylome, only 21 proteins were significantly regulated by chronic exercise. Relatively little change of proteins in brain have also been reported under several other conditions, like C. neoformans infection (Li et al., 2020) and epilepsy (Qian et al., 2022). Therefore, acetyl modification of proteins is likely more sensitive to internal or external stimuli than the protein *per se*, and might provide new insights into exercise physiology.

Acetylation is regulated by KATs and KDACs. Under pathological stress, chronic exercise can improve cognitive function by activating SIRT1 or SIRT3, whereas Koltai et al. (2011) reported no difference of SIRT1 expression between young and old rats. We screened out several KATs and KDACs, but there was no difference between Ex and Con groups (Supplementary Table 7). SIRT3, the major deacetylase in mitochondria, might increase during exercising as one of its major targets ACAT1 was downregulated. Deacetylation might have taken place during or shortly after each training session because of fluctuations in acetyl-CoA and NAD^+^ levels as well as temporary imbalance of KATs/KDACs. And 24 h lagging behind last running allowed the body to recover its homeostasis, including the KAT/KDAC balance in healthy wild-type mice.

There were several limitations in the present study. First, three biological replicates with one model of exercise were used to characterize acetylation map in hippocampus. Despite bilateral hippocampus from every two mice were mixed as one replicate to reduce the biological variation, it’s necessary to verify the results with more samples. Likewise, other models like resistance exercise or high-intensity interval training are worth being explored to unmask common or model-specific acetylation maps. Second, as subfields of the hippocampus (area CA1, area CA3 and DG) may operate differently in memory formation, sampling of whole hippocampus in our study likely sheltered some information. More accurate evidence is appreciated to describe protein acetylation in each subfield of hippocampus in response to exercise. Third, alterations of the acetylated proteins identified in the present study could not be experimentally verified due to a lack of commercially available antibodies for the specific sites, and we also lack mechanism-focused studies for key acetylated proteins like 14-3-3 gamma. We will focus on functional studies in the future.

In conclusion, our study generated a molecular map of acetylation in the hippocampus in response to moderate-intensity chronic exercise, partially revealing the molecular mechanisms underlying the benefits of exercise on hippocampal function. Exercise significantly changed 272 acetyl sites on 252 proteins, with 18.58% acetylated proteins annotated in mitochondria. These proteins were dominantly deacetylated and closely related to carbon-related metabolism, the Hippo signaling pathway, ribosomes and protein processing. Meanwhile, 21 proteins were significantly expressed, which were enriched in the pathway of complement and coagulation cascades. Our study provides a new avenue to help understand the molecular mechanisms underpinning the benefits of exercise for brain health. Our current knowledge of lysine acetylation in the life activity is just the tip of the iceberg, future research in the field of exercise biology is required to clarify how acetylation affects the critical nodes of certain pathways to and benefits the brain and other systems under various situations.

## Data availability statement

Acetylomic and proteomic data are deposited in public databases: ProteomeXchange, (PXD034452). Further inquiries can be directed to the corresponding authors upon reasonable request.

## Ethics statement

The animal study was reviewed and approved by the Ethics Committee on Animal Care and Use of the Capital Institute of Pediatrics, Beijing, China (DWLL2021015).

## Author contributions

PQ, TZ, SW, and JW designed the study and drafted the manuscript. PQ, FM, and WZ carried out the mouse study. PQ, DC, LL, ZL, PP, TZ, SW, and JW contributed to the data acquisition, interpretation, and discussion. All authors contributed to the final version of the manuscript.

## Footnotes

1
https://biit.cs.ut.ee/clustvis/

2
https://wolfpsort.hgc.jp/

3
http://www.ebi.ac.uk/interpro/

## Supplementary material

Supplementary Figure 1 Running protocol and physical response to chronic exercise, related to Figure 1. **(A)** Running protocol in acclimation stage. **(B,C)** physical response to chronic exercise (*n* = 11–12). Compared with the sedentary control, chronic exercise significantly enhanced the relative mass of vastus lateralis (4.28 ± 0.18 vs. 5.11 ± 0.10, *p* = 0.0004), and suppressed epididymal fat gains (9.59 ± 0.68 vs. 7.71 ± 0.42, *p* = 0.0282). Data are presented as mean ± SEM, **p* < 0.05, ****p* < 0.001 Ex vs. Con by unpaired Student’s *t*-test. Con, control; Ex, exercise.

Supplementary Figure 2 Distribution of mass error for the identified acetylated peptides.

Supplementary Figure 3 Subcellular distribution predicted for the 252 differentially acetylated proteins.

Supplementary Figure 4 Kyoto Encyclopedia of Genes and Genomes (KEGG) pathway analysis for 252 differentially acetylated proteins.

Supplementary Figure 5 Acetylated proteins and sites in significantly enriched pathways of glycolysis/gluconeogenesis **(A)** and Hippo signaling **(B)**.

## References

[B1] Aebersold R. Agar J. N. Amster I. J. Baker M. S. Bertozzi C. R. Boja E. S. (2018). How many human proteoforms are there? *Nat. Chem. Biol.* 14 206–214. 10.1038/Nchembio.2576 29443976 PMC5837046

[B2] Aghazadeh Y. Ye X. Blonder J. Papadopoulos V. (2014). Protein modifications regulate the role of 14-3-3gamma adaptor protein in cAMP-induced steroidogenesis in MA-10 Leydig cells. *J. Biol. Chem.* 289 26542–26553. 10.1074/jbc.M114.569079 25086053 PMC4176220

[B3] Antunes A. S. L. M. Saia-Cereda V. M. Crunfli F. Martins-de-Souza D. (2022). 14-3-3 proteins at the crossroads of neurodevelopment and schizophrenia. *World J. Biol. Psychiatry* 23 14–32. 10.1080/15622975.2021.1925585 33952049

[B4] Baeza J. Smallegan M. J. Denu J. M. (2016). Mechanisms and dynamics of protein acetylation in mitochondria. *Trends Biochem. Sci.* 41 231–244. 10.1016/j.tibs.2015.12.006 26822488 PMC4783225

[B5] Bo H. Kang W. Jiang N. Wang X. Zhang Y. Ji L. L. (2014). Exercise-induced neuroprotection of hippocampus in APP/PS1 transgenic mice via upregulation of mitochondrial 8-oxoguanine DNA glycosylase. *Oxid Med. Cell Longev.* 2014:834502. 10.1155/2014/834502 25538817 PMC4236906

[B6] Cheng J. B. Wang S. K. Dong Y. Yuan Z. Q. (2020). The role and regulatory mechanism of hippo signaling components in the neuronal system. *Front. Immunol.* 11:281. 10.3389/fimmu.2020.00281 32140159 PMC7042394

[B7] Choudhary C. Kumar C. Gnad F. Nielsen M. L. Rehman M. Walther T. C. (2009). Lysine acetylation targets protein complexes and co-regulates major cellular functions. *Science* 325 834–840. 10.1126/science.1175371 19608861

[B8] Chrysostomou V. Galic S. van Wijngaarden P. Trounce I. A. Steinberg G. R. Crowston J. G. (2016). Exercise reverses age-related vulnerability of the retina to injury by preventing complement-mediated synapse elimination via a BDNF-dependent pathway. *Aging Cell* 15:1082–1091. 10.1111/acel.12512 27613664 PMC5114604

[B9] Colaert N. Helsens K. Martens L. Vandekerckhove J. Gevaert K. (2009). Improved visualization of protein consensus sequences by iceLogo. *Nat. Methods* 6 786–787. 10.1038/nmeth1109-786 19876014

[B10] Contrepois K. Wu S. Moneghetti K. J. Hornburg D. Ahadi S. Tsai M. S. (2020). Molecular choreography of acute exercise. *Cell* 181:e1116. 10.1016/j.cell.2020.04.043 32470399 PMC7299174

[B11] Cooper C. Moon H. Y. van Praag H. (2018). On the run for hippocampal plasticity. *Cold Spring Harb. Perspect. Med.* 8:a029736. 10.1101/cshperspect.a029736 28495803 PMC5880155

[B12] de Meireles L. C. F. Galvao F. Jr. Walker D. M. Cechinel L. R. de Souza Grefenhagen A. I. Andrade G. (2019). Exercise modalities improve aversive memory and survival rate in aged rats: Role of hippocampal epigenetic modifications. *Mol. Neurobiol.* 56 8408–8419. 10.1007/s12035-019-01675-w 31250382 PMC6918477

[B13] De Miguel Z. Khoury N. Betley M. J. Lehallier B. Willoughby D. Olsson N. (2021). Exercise plasma boosts memory and dampens brain inflammation via clusterin. *Nature* 600 494–499. 10.1038/s41586-021-04183-x 34880498 PMC9721468

[B14] Ding D. Mutrie N. Bauman A. Pratt M. Hallal P. R. C. Powell K. E. (2020). Physical activity guidelines 2020: Comprehensive and inclusive recommendations to activate populations. *Lancet* 396 1780–1782. 10.1016/S0140-6736(20)32229-7 33248019

[B15] Duzel E. van Praag H. Sendtner M. (2016). Can physical exercise in old age improve memory and hippocampal function? *Brain* 139 662–673. 10.1093/brain/awv407 26912638 PMC4766381

[B16] Erickson K. I. Leckie R. L. Weinstein A. M. (2014). Physical activity, fitness, and gray matter volume. *Neurobiol. Aging* 35 S20–S28. 10.1016/j.neurobiolaging.2014.03.034 24952993 PMC4094356

[B17] Erickson K. I. Voss M. W. Prakash R. S. Basak C. Szabo A. Chaddock L. (2011). Exercise training increases size of hippocampus and improves memory. *Proc. Natl. Acad. Sci. U.S.A.* 108 3017–3022. 10.1073/pnas.1015950108 21282661 PMC3041121

[B18] Fernando P. Bonen A. Hoffman-Goetz L. (1993). Predicting submaximal oxygen consumption during treadmill running in mice. *Can. J. Physiol. Pharmacol.* 71 854–857. 10.1139/y93-128 8143245

[B19] Finley L. W. Carracedo A. Lee J. Souza A. Egia A. Zhang J. (2011). SIRT3 opposes reprogramming of cancer cell metabolism through HIF1alpha destabilization. *Cancer Cell* 19 416–428. 10.1016/j.ccr.2011.02.014 21397863 PMC3065720

[B20] Frontini-Lopez Y. R. Gojanovich A. D. Del Veliz S. Uhart M. Bustos D. M. (2021). 14-3-3beta isoform is specifically acetylated at Lys51 during differentiation to the osteogenic lineage. *J. Cell Biochem.* 122 1767–1780. 10.1002/jcb.30128 34379822

[B21] Fu V. Plouffe S. W. Guan K. L. (2017). The Hippo pathway in organ development, homeostasis, and regeneration. *Curr. Opin. Cell Biol.* 49 99–107. 10.1016/j.ceb.2017.12.012 29316535 PMC6348871

[B22] Haapalainen A. M. Merilainen G. Pirila P. L. Kondo N. Fukao T. Wierenga R. K. (2007). Crystallographic and kinetic studies of human mitochondrial acetoacetyl-CoA thiolase: The importance of potassium and chloride ions for its structure and function. *Biochemistry* 46 4305–4321. 10.1021/bi6026192 17371050

[B23] Hebert A. S. Dittenhafer-Reed K. E. Yu W. Bailey D. J. Selen E. S. Boersma M. D. (2013). Calorie restriction and SIRT3 trigger global reprogramming of the mitochondrial protein acetylome. *Mol. Cell* 49 186–199. 10.1016/j.molcel.2012.10.024 23201123 PMC3704155

[B24] Hirschey M. D. Shimazu T. Jing E. Grueter C. A. Collins A. M. Aouizerat B. (2011). SIRT3 deficiency and mitochondrial protein hyperacetylation accelerate the development of the metabolic syndrome. *Mol. Cell* 44 177–190. 10.1016/j.molcel.2011.07.019 21856199 PMC3563434

[B25] Ivanov G. S. Ivanova T. Kurash J. Ivanov A. Chuikov S. Gizatullin F. (2007). Methylation-acetylation interplay activates p53 in response to DNA damage. *Mol. Cell Biol.* 27 6756–6769. 10.1128/MCB.00460-07 17646389 PMC2099237

[B26] Kim S. C. Sprung R. Chen Y. Xu Y. Ball H. Pei J. (2006). Substrate and functional diversity of lysine acetylation revealed by a proteomics survey. *Mol. Cell* 23 607–618. 10.1016/j.molcel.2006.06.026 16916647

[B27] Kjaer M. Secher N. H. Galbo H. (1987). Physical stress and catecholamine release. *Baillieres Clin. Endocrinol. Metab.* 1 279–298. 10.1016/s0950-351x(87)80064-2 3327495

[B28] Koltai E. Zhao Z. Lacza Z. Cselenyak A. Vacz G. Nyakas C. (2011). Combined exercise and insulin-like growth factor-1 supplementation induces neurogenesis in old rats, but do not attenuate age-associated DNA damage. *Rejuvenation Res.* 14 585–596. 10.1089/rej.2011.1178 21867412 PMC3239321

[B29] Li H. Li Y. Sun T. Du W. Zhang Z. Li D. (2020). Integrative proteome and acetylome analyses of murine responses to cryptococcus neoformans infection. *Front. Microbiol.* 11:575. 10.3389/fmicb.2020.00575 32362878 PMC7181412

[B30] Li T. T. Liu M. X. Feng X. Wang Z. Das I. Xu Y. P. (2014). Glyceraldehyde-3-phosphate dehydrogenase is activated by lysine 254 acetylation in response to glucose signal. *J. Biol. Chem.* 289 3775–3785. 10.1074/jbc.M113.531640 24362262 PMC3916574

[B31] Lin M. T. Beal M. F. (2006). Mitochondrial dysfunction and oxidative stress in neurodegenerative diseases. *Nature* 443 787–795. 10.1038/nature05292 17051205

[B32] Liu L. Wang H. Rao X. Yu Y. Li W. Zheng P. (2021). Comprehensive analysis of the lysine acetylome and succinylome in the hippocampus of gut microbiota-dysbiosis mice. *J. Adv. Res.* 30 27–38. 10.1016/j.jare.2020.12.002 34026284 PMC8132208

[B33] Lundby A. Lage K. Weinert B. T. Bekker-Jensen D. B. Secher A. Skovgaard T. (2012). Proteomic analysis of lysine acetylation sites in rat tissues reveals organ specificity and subcellular patterns. *Cell Rep.* 2 419–431. 10.1016/j.celrep.2012.07.006 22902405 PMC4103158

[B34] Maass A. Duzel S. Goerke M. Becke A. Sobieray U. Neumann K. (2015). Vascular hippocampal plasticity after aerobic exercise in older adults. *Mol. Psychiatry* 20 585–593. 10.1038/mp.2014.114 25311366

[B35] Marlatt M. W. Potter M. C. Lucassen P. J. van Praag H. (2012). Running throughout middle-age improves memory function, hippocampal neurogenesis, and BDNF levels in female C57BL/6J mice. *Dev. Neurobiol.* 72 943–952. 10.1002/dneu.22009 22252978 PMC3485396

[B36] Min S. W. Chen X. Tracy T. E. Li Y. Q. Zhou Y. G. Wang C. (2015). Critical role of acetylation in tau-mediated neurodegeneration and cognitive deficits. *Nat. Med.* 21 1154–1162. 10.1038/nm.3951 26390242 PMC4598295

[B37] Nakayasu E. S. Burnet M. C. Walukiewicz H. E. Wilkins C. S. Shukla A. K. Brooks S. (2017). Ancient regulatory role of lysine acetylation in central metabolism. *mBio* 8 e1894–e1817. 10.1128/mBio.01894-17 29184018 PMC5705920

[B38] Narita T. Weinert B. T. Choudhary C. (2019). Functions and mechanisms of non-histone protein acetylation. *Nat. Rev. Mol. Cell Biol.* 20 156–174. 10.1038/s41580-018-0081-3 30467427

[B39] Nay K. Smiles W. J. Kaiser J. McAloon L. M. Loh K. Galic S. (2021). Molecular mechanisms underlying the beneficial effects of exercise on brain function and neurological disorders. *Int. J. Mol. Sci.* 22:4052. 10.3390/ijms22084052 33919972 PMC8070923

[B40] O’Callaghan R. M. Griffin E. W. Kelly A. M. (2009). Long-term treadmill exposure protects against age-related neurodegenerative change in the rat hippocampus. *Hippocampus* 19 1019–1029. 10.1002/hipo.20591 19309034

[B41] Ouidir T. Kentache T. Hardouin J. (2016). Protein lysine acetylation in bacteria: Current state of the art. *Proteomics* 16 301–309. 10.1002/pmic.201500258 26390373

[B42] Prakash R. S. Voss M. W. Erickson K. I. Kramer A. F. (2015). Physical activity and cognitive vitality. *Annu. Rev. Psychol.* 66 769–797. 10.1146/annurev-psych-010814-015249 25251492

[B43] Qian X. Ding J. Q. Zhao X. Sheng X. W. Wang Z. R. Yang Q. X. (2022). Proteomic analysis reveals the vital role of synaptic plasticity in the pathogenesis of temporal lobe epilepsy. *Neural Plast.* 2022:8511066. 10.1155/2022/8511066 35860309 PMC9293557

[B44] Ringholm S. Gudiksen A. Halling J. F. Qoqaj A. Rasmussen P. M. Prats C. (2022). Impact of aging and lifelong exercise training on mitochondrial function and network connectivity in human skeletal muscle. *J. Gerontol. A Biol. Sci. Med. Sci.* 10.1093/gerona/glac164 [Epub ahead of print]. 35961318

[B45] Sabari B. R. Zhang D. Allis C. D. Zhao Y. (2017). Metabolic regulation of gene expression through histone acylations. *Nat. Rev. Mol. Cell Biol.* 18 90–101. 10.1038/nrm.2016.140 27924077 PMC5320945

[B46] Salomon D. Orth K. (2013). What pathogens have taught us about posttranslational modifications. *Cell Host Microbe* 14 269–279. 10.1016/j.chom.2013.07.008 24034613 PMC5785091

[B47] Sanford J. A. Nogiec C. D. Lindholm M. E. Adkins J. N. Amar D. Dasari S. (2020). Molecular transducers of physical activity consortium (MoTrPAC): Mapping the dynamic responses to exercise. *Cell* 181 1464–1474. 10.1016/j.cell.2020.06.004 32589957 PMC8800485

[B48] Sant’Anna M. L. Oliveira L. T. Gomes D. V. Marques S. T. F. Provance D. W. Jr. Sorenson M. M. (2019). Physical exercise stimulates salivary secretion of cystatins. *PLoS One* 14:e0224147. 10.1371/journal.pone.0224147 31648256 PMC6874361

[B49] Sarga L. Hart N. Koch L. G. Britton S. L. Hajas G. Boldogh I. (2013). Aerobic endurance capacity affects spatial memory and SIRT1 is a potent modulator of 8-oxoguanine repair. *Neuroscience* 252 326–336. 10.1016/j.neuroscience.2013.08.020 23973402 PMC3856236

[B50] Sato S. Dyar K. A. Treebak J. T. Jepsen S. L. Ehrlich A. M. Ashcroft S. P. (2022). Atlas of exercise metabolism reveals time-dependent signatures of metabolic homeostasis. *Cell Metab.* 34:e328. 10.1016/j.cmet.2021.12.016 35030324 PMC13189211

[B51] Sun L. Bhawal R. Xu H. Chen H. Anderson E. T. Haroutunian V. (2021). The human brain acetylome reveals that decreased acetylation of mitochondrial proteins associates with Alzheimer’s disease. *J. Neurochem.* 158 282–296. 10.1111/jnc.15377 33905124

[B52] Toth Z. E. Mezey E. (2007). Simultaneous visualization of multiple antigens with tyramide signal amplification using antibodies from the same species. *J. Histochem. Cytochem.* 55 545–554. 10.1369/jhc.6A7134.2007 17242468

[B53] Urdinguio R. G. Tejedor J. R. Fernandez-Sanjurjo M. Perez R. F. Penarroya A. Ferrero C. (2021). Physical exercise shapes the mouse brain epigenome. *Mol. Metab.* 54:101398. 10.1016/j.molmet.2021.101398 34801767 PMC8661702

[B54] Wagner S. A. Beli P. Weinert B. T. Nielsen M. L. Cox J. Mann M. (2011). A proteome-wide, quantitative survey of in vivo ubiquitylation sites reveals widespread regulatory roles. *Mol. Cell Proteomics* 10:M111013284. 10.1074/mcp.M111.013284 21890473 PMC3205876

[B55] Walsh C. T. Garneau-Tsodikova S. Gatto G. J. Jr. (2005). Protein posttranslational modifications: The chemistry of proteome diversifications. *Angew. Chem. Int. Ed Engl.* 44 7342–7372. 10.1002/anie.200501023 16267872

[B56] Wang Q. Zhang Y. Yang C. Xiong H. Lin Y. Yao J. (2010). Acetylation of metabolic enzymes coordinates carbon source utilization and metabolic flux. *Science* 327 1004–1007. 10.1126/science.1179687 20167787 PMC4183141

[B57] Wang Z. Zheng R. Wang X. Huang X. Huang J. Gu C. (2022). Aerobic exercise improves methamphetamine-induced olfactory dysfunction through alpha-synuclein intervention in male mice. *Front. Mol. Neurosci.* 15:884790. 10.3389/fnmol.2022.884790 35586307 PMC9108672

[B58] Whiteman A. S. Young D. E. Budson A. E. Stern C. E. Schon K. (2016). Entorhinal volume, aerobic fitness, and recognition memory in healthy young adults: A voxel-based morphometry study. *Neuroimage* 126 229–238. 10.1016/j.neuroimage.2015.11.049 26631814 PMC4733633

[B59] Xu H. D. Zhou J. Q. Lin S. F. Deng W. K. Zhang Y. Xue Y. (2017). PLMD: An updated data resource of protein lysine modifications. *J. Genet. Genomics* 44 243–250. 10.1016/j.jgg.2017.03.007 28529077

[B60] Yang L. Vaitheesvaran B. Hartil K. Robinson A. J. Hoopmann M. R. Eng J. K. (2011). The fasted/fed mouse metabolic acetylome: N6-acetylation differences suggest acetylation coordinates organ-specific fuel switching. *J. Proteome Res.* 10 4134–4149. 10.1021/pr200313x 21728379 PMC3204869

[B61] Zhang H. L. Zhao B. Han W. Sun Y. B. Yang P. Chen Y. (2021). Acetylation of calmodulin regulates synaptic plasticity and fear learning. *J. Biol. Chem.* 297:101034. 10.1016/j.jbc.2021.101034 34339735 PMC8383114

[B62] Zhang Y. Zhou F. Bai M. Liu Y. Zhang L. Zhu Q. (2019). The pivotal role of protein acetylation in linking glucose and fatty acid metabolism to beta-cell function. *Cell Death Dis.* 10:66. 10.1038/s41419-019-1349-z 30683850 PMC6347623

[B63] Zhong T. Ren F. Huang C. S. Zou W. Y. Yang Y. Pan Y. D. (2016). Swimming exercise ameliorates neurocognitive impairment induced by neonatal exposure to isoflurane and enhances hippocampal histone acetylation in mice. *Neuroscience* 316 378–388. 10.1016/j.neuroscience.2015.12.049 26748054

