# Supplementary figures and images for: Chronic exercise remodels the lysine acetylome in the mouse hippocampus
Source: Front Mol Neurosci. 2022 Oct 28;15:1023482. doi: 10.3389/fnmol.2022.1023482 (PMC9650339; doi:10.3389/fnmol.2022.1023482)

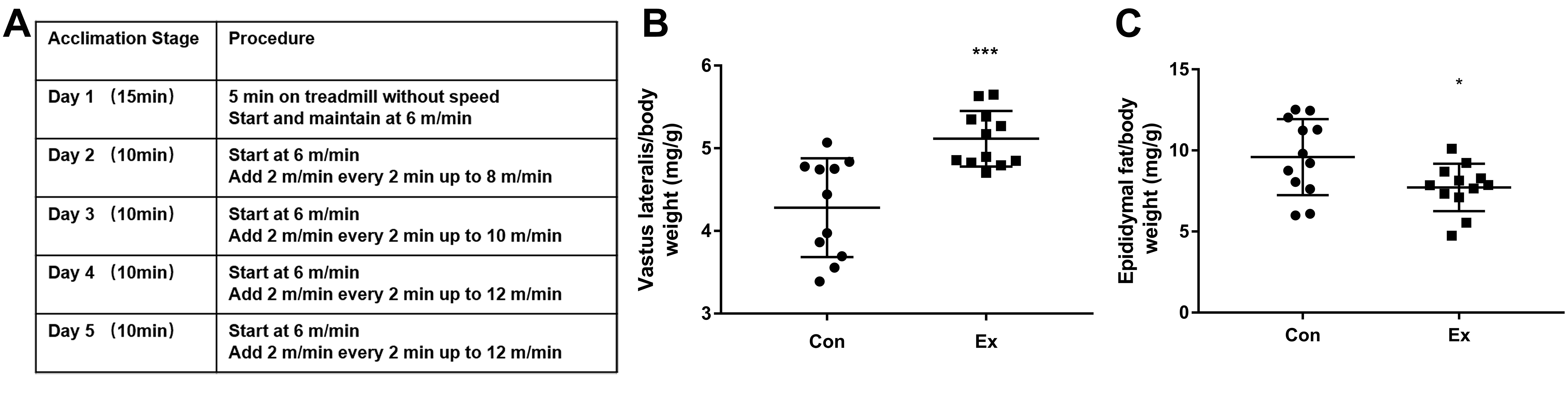

Supplement: Supplementary Figure 1 — Running protocol and physical response to chronic exercise, related to Figure 1. (A) Running protocol in acclimation stage. (B,C) physical response to chronic exercise (n = 11–12). Compared with the sedentary control, chronic exercise significantly enhanced the relative mass of vastus lateralis (4.28 ± 0.18 vs. 5.11 ± 0.10, p = 0.0004), and suppressed epididymal fat gains (9.59 ± 0.68 vs. 7.71 ± 0.42, p = 0.0282). Data are presented as mean ± SEM, *p < 0.05, ***p < 0.001 Ex vs. Con by unpaired Student’s t-test. Con, control; Ex, exercise. [file Image_1.TIF]

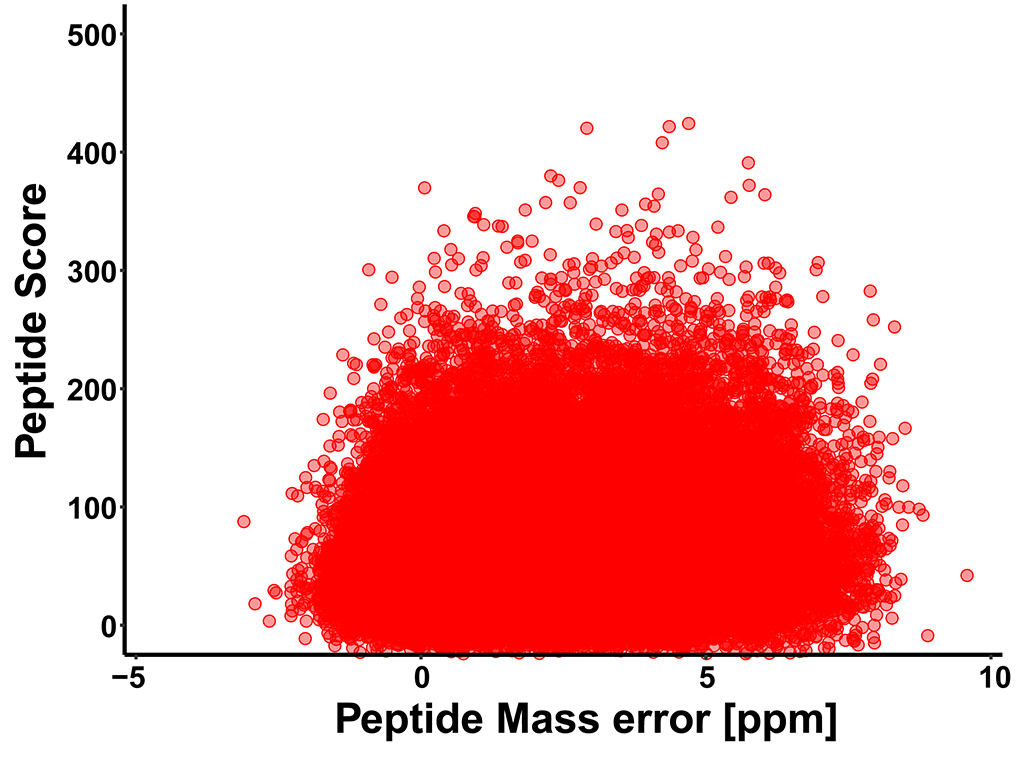

Supplement: Supplementary Figure 2 — Distribution of mass error for the identified acetylated peptides. [file Image_2.TIF]

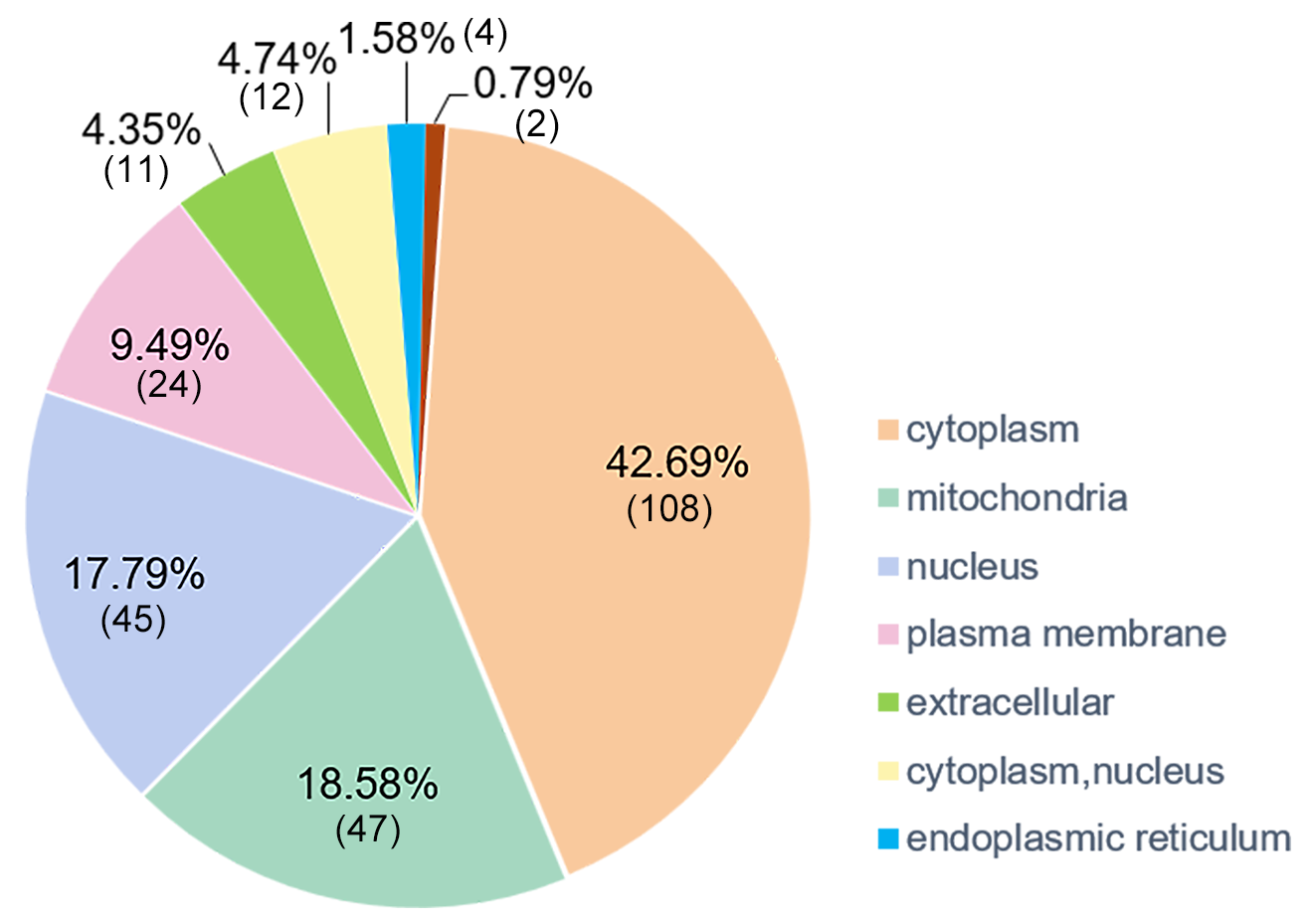

Supplement: Supplementary Figure 3 — Subcellular distribution predicted for the 252 differentially acetylated proteins. [file Image_3.TIF]

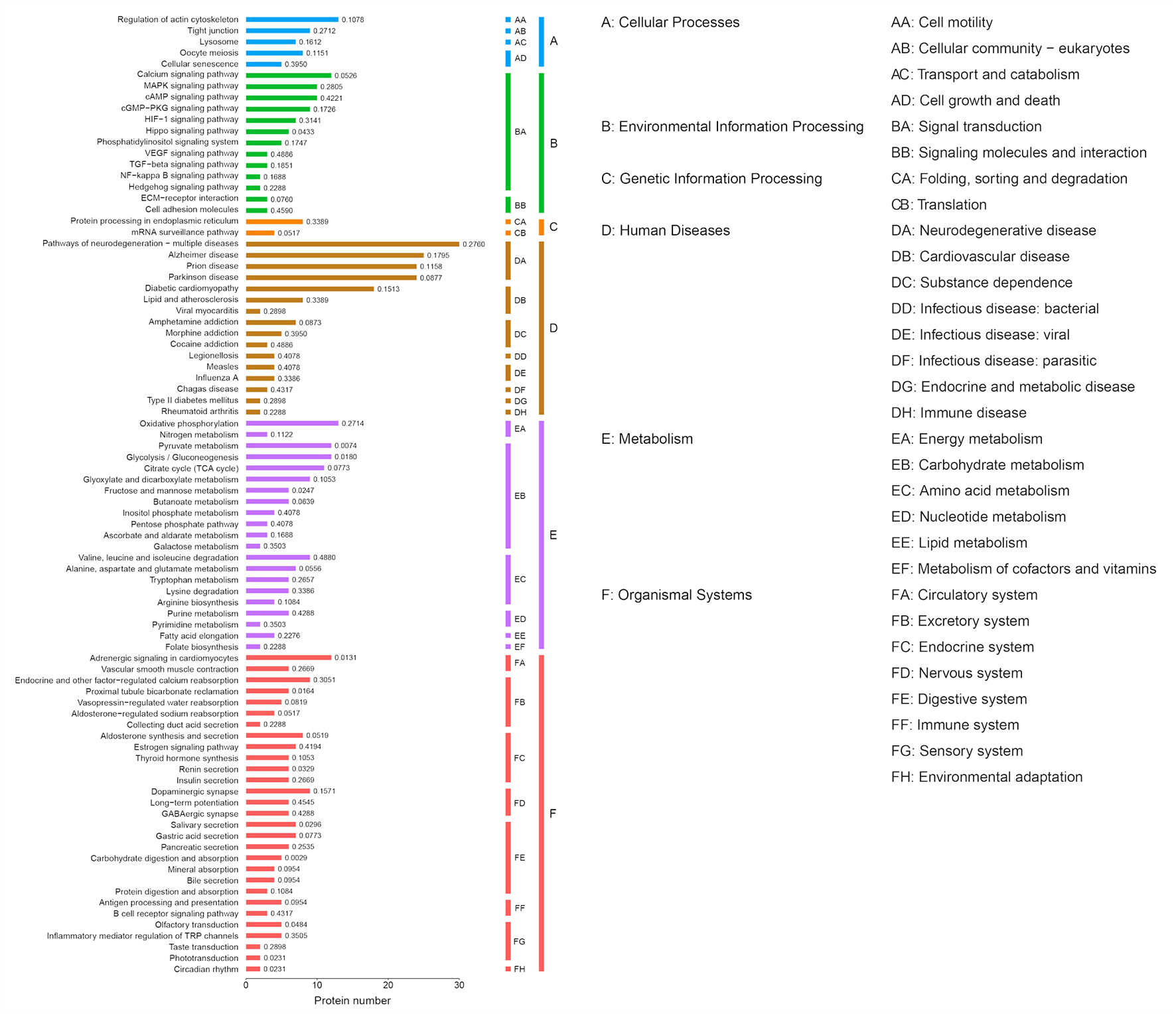

Supplement: Supplementary Figure 4 — Kyoto Encyclopedia of Genes and Genomes (KEGG) pathway analysis for 252 differentially acetylated proteins. [file Image_4.TIF]

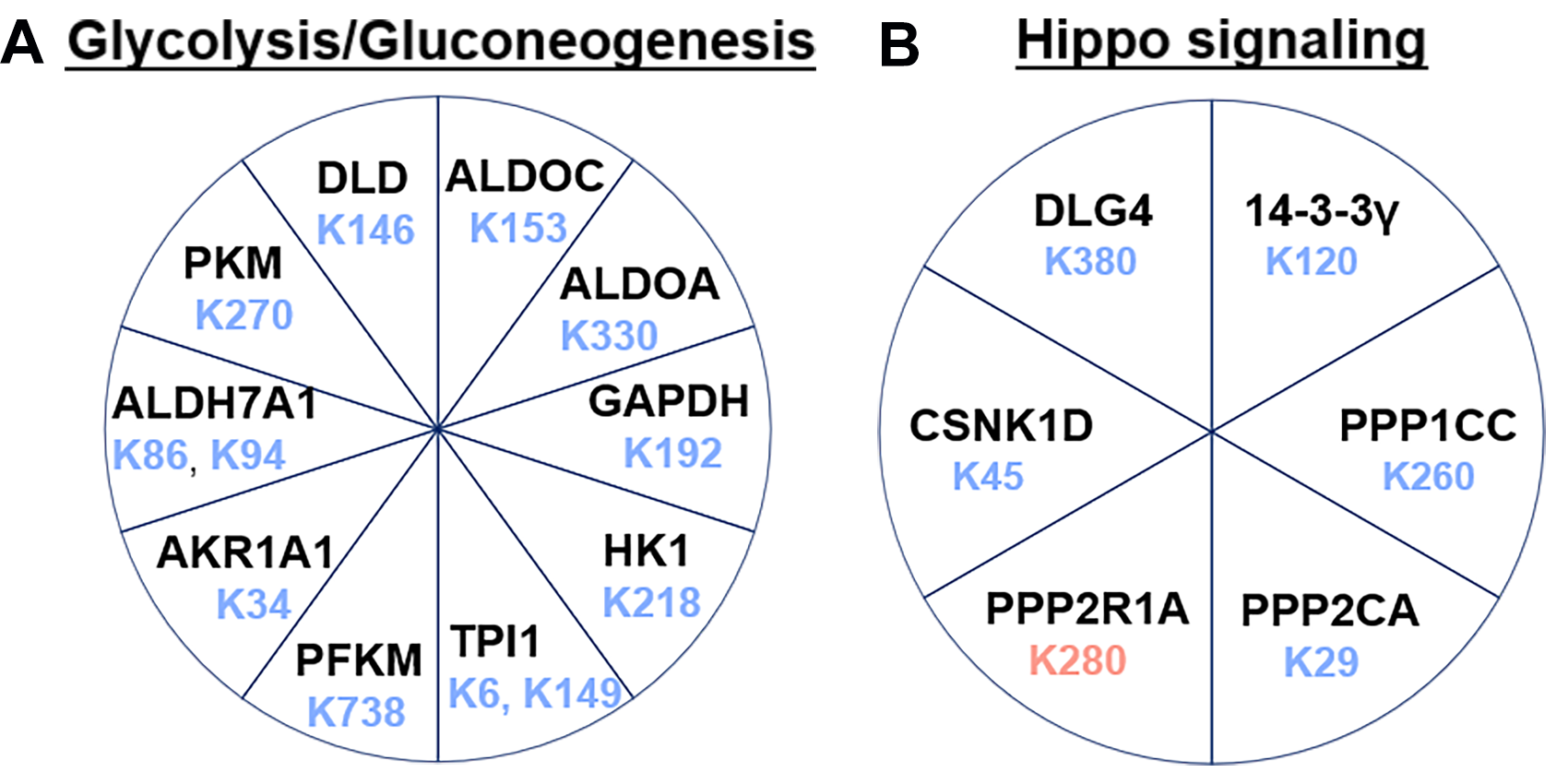

Supplement: Supplementary Figure 5 — Acetylated proteins and sites in significantly enriched pathways of glycolysis/gluconeogenesis (A) and Hippo signaling (B). [file Image_5.TIF]
